# Salt reduction policy for out of home sectors: a supplementary document for the salt reduction strategy to prevent and control non-communicable diseases (NCDS) in Malaysia 2021–2025

**DOI:** 10.1186/s12961-024-01124-8

**Published:** 2024-04-18

**Authors:** Zaliha Harun, Suzana Shahar, Yee Xing You, Zahara Abdul Manaf, Hazreen Abdul Majid, Chia Yook Chin, Hasnah Haron, Viola Michael, Hamdan Mohamad, Siti Farrah Zaidah Mohd Yazid, Musaalbakri Abdul Manan, Wan Zunairah Wan Ibadullah, Mhairi K. Brown, Feng J. He, Graham A. MacGregor

**Affiliations:** 1 Dietetic Programme, Centre for Healthy Ageing and Wellness (H-Care), Faculty of Health Sciences, Universiti Kebangsaan Malaysia, Jalan Raja Muda Abdul Aziz, 50300 Kuala Lumpur, Malaysia; 2 Centre for Population Health, Department of Social and Preventive Medicine, Faculty of Medicine, University of Malaya, 50603 Kuala Lumpur, Malaysia; 3 Department of Medical Sciences, School of Medical and Life Sciences, Sunway University, 47500 Subang Jaya, Selangor Darul Ehsan Malaysia; 4 Department of Primary Care Medicine, Faculty of Medicine, University of Malaya, 50603 Kuala Lumpur, Malaysia; 5 Nutritional Sciences Programme, Centre for Healthy Ageing and Wellness (H-Care), Faculty of Health Sciences, Universiti Kebangsaan Malaysia, Jalan Raja Muda Abdul Aziz, 50300 Kuala Lumpur, Malaysia; 6 Ministry of Health (MOH), Block A, Chancery Place, Precinct 15, 62050 Putrajaya, Malaysia; 7 Ministry of Health (MOH), Block E3, Complex E, Federal Government Administrative Centre, 62590 Putrajaya, Malaysia; 8 Food Science and Technology Research Centre, Malaysia Agricultural Research and Development Institute, Serdang, Selangor Malaysia; 9 Faculty of Science and Food Technology, Universiti Putra Malaysia, Serdang, Selangor Malaysia; 10 Wolfson Institute of Population Health, Barts and The London School of Medicine and Dentistry, Queen Mary University of London, London, EC1M 6BQ United Kingdom; 11 School of Health and Rehabilitation Sciences, AECC University College, Bournemouth, BH5 2DF United Kingdom; 12 Lincoln University College—Medical Campus, 2, Jalan Stadium SS 7/15, Ss 7, 47301 Petaling Jaya, Selangor Malaysia

**Keywords:** Salt reduction policy, Out-of-home sectors, Supplementary document, Policy-makers, Food industries, Food operators, School, Malaysia

## Abstract

Cardiovascular diseases (CVDs) are the major cause of death among Malaysians. Reduction of salt intake in populations is one of the most cost-effective strategies in the prevention of CVDs. It is very feasible as it requires low cost for implementation and yet could produce a positive impact on health. Thus, salt reduction initiatives have been initiated since 2010, and two series of strategies have been launched. However, there are issues on its delivery and outreach to the target audience. Further, strategies targeting out of home sectors are yet to be emphasized. Our recent findings on the perceptions, barriers and enablers towards salt reduction among various stakeholders including policy-makers, food industries, food operators, consumers and schools showed that eating outside of the home contributed to high salt intake. Foods sold outside the home generally contain a high amount of salt. Thus, this supplementary document is being proposed to strengthen the Salt Reduction Strategy to Prevent and Control Non-communicable Diseases (NCDs) for Malaysia 2021–2025 by focussing on the strategy for the out-of-home sectors. In this supplementary document, the Monitoring, Awareness and Product (M-A-P) strategies being used by the Ministry of Health (MOH) are adopted with a defined outline of the plan of action and indicators to ensure that targets could be achieved. The strategies will involve inter-sectoral and multi-disciplinary approaches, including monitoring of salt intake and educating consumers, strengthening the current enforcement of legislation on salt/sodium labelling and promoting research on reformulation. Other strategies included in this supplementary document included reformulation through proposing maximum salt targets for 14 food categories. It is hoped that this supplementary document could strengthen the current the Salt Reduction Strategy to Prevent and Control NCDs for Malaysia 2021–2025 particularly, for the out-of-home sector, to achieve a reduction in mean salt intake of the population to 6.0 g per day by 2025.

## Introduction

Salt is the main source of sodium in the diet. In most developed countries, high salt intake is related to the regular consumption of processed foods, while in low- and middle-income countries (LMIC), high salt intake is related to discretional salt used during cooking and through condiments such as fish sauce and soy sauce [1]. In addition, the outside foods sold by street vendors or at other food premises also have a high salt content [2]. High salt intake is associated with elevated blood pressure, a risk factor for cardiovascular diseases (CVDs) [3]. Currently, the WHO recommends a maximum of 5 g of salt per day [4]. It is estimated that the vast majority of adults (99.2%) worldwide consume more than the recommended salt intake by WHO [5]. Globally, about 1.28 billion adults aged 30–79 years old have hypertension, with two thirds living in LMICs, including Malaysia [6]. Increasing trend of prevalence of hypertension has also been recorded in LMICs, especially in Sub-Saharan Africa, the Middle East and North Africa, East Asia and the Pacific during the last three decades [7].

Salt reduction has been identified as one of the most cost-effective strategies to prevent non-communicable diseases (NCDs). Salt intake of less than 5 g per day for adults helps to reduce blood pressure and the risk of CVDS [4]. Thus, an interim target of 30% reduction in population salt intake has been included as one of nine targets set in the World Action Plan on Prevention and Control of NCDs 2013–2020. This plan aimed to achieve a 25% reduction of premature death from NCDs including CVDs by 2025 [8]. Many countries around the world are working towards the reduction of salt intake in the population. Approximately 96 countries now have national salt-reduction strategies in place. Most of these salt reduction strategies are multi-faceted. These strategies included intervention in different settings (i.e. schools, workplaces, hospitals, government offices), food reformulation (i.e. voluntary salt target or mandatory maximum salt limits), consumer education, front-of-pack labelling and taxation [9].

### Salt intake in Malaysia

According to the Malaysian Community Salt Survey (MyCoSS 2019) [10] conducted between October 2017 and March 2018, Malaysian adults consumed an average of 7.9 g salt per day, with 79% with an intake more than the WHO’s recommended level of 5 g/day (sodium 2000 mg/day). This exposes the nation to the risk of developing hypertension and CVDs [10]. The National Health & Morbidity Survey reported that one in three Malaysian adults had hypertension (NHMS 2019) [11]. The MyCoSS study also found that among the most consumed foods with high sodium were fried vegetables, bread and soy sauce. The highest content of sodium has been reported in *kolok mee*, followed by light soy sauce and curry noodles [10]. Market surveys showed that half of the high salt content products such as soy sauce and instant noodles did not have sodium/salt labelling [12, 13]. A recent survey of street foods reported that snack food category contained the highest amount of sodium, followed by main meals and desserts. The snack food category included processed foods (e.g. fried fish balls, fried chicken with cheese, fried crab meatballs, fried sausages and fried chicken balls), fish crackers (*keropok lekor*) and seaweed pickles. Main dishes such as fried noodles, noodles with gravy, fried rice and other cooked rice also contain medium to high amounts of sodium. Noodle-based dishes were reported to contain more than 2000 mg of sodium per serving; and together with other cooked dishes were the most consumed street food group [2].

### Salt reduction policy in Malaysia

**Fig. 1:**
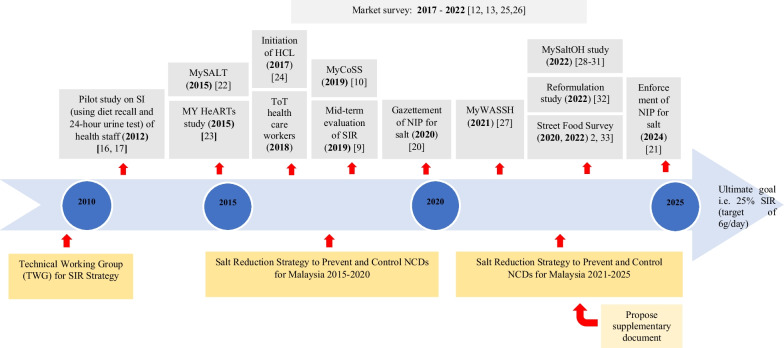
Timeline of the salt reduction policy in Malaysia. MySALT 2015: dietary sodium intake among the Ministry of Health staff 2015 [16]. MY HeARTs Study 2015: Malaysian Health and Adolescents Longitudinal Research Team Study 2015[17]. MyCoSS 2019: Malaysian Community Salt Survey 2019 [10]. Newton study: Developing a policy to reduce the salt content of food consumed outside the home in Malaysia [20]. Resolve study: Development of Salt Reduction Target for Malaysia [27]

Historically, the salt reduction strategy was initiated in 2010 by the salt reduction committee under the Non-Communicable Disease (NCD) Section of the Disease Control Division, Ministry of Health (MOH) [14]. Figure 1 outlined the timeline of the salt reduction policy in Malaysia. A technical working group (TWG) was formed and published the Salt Reduction Strategy to Prevent and Control NCDs for Malaysia 2015–2020 [14]. The policy has been further revised for its second version for 2021–2025 [15]. This salt reduction policy is part of the National Strategic Plan for Non-Communicable Disease (NSP–NCD) 2016–2025 [14]. Generally, the objectives of the salt reduction policy are to promote, educate and collaborate with all stakeholders to reduce population salt intake by 30% from 8.7 g/day to 6.0 g/day by the year 2025. The baseline level of 8.7 g/day is based on an earlier study among healthcare workers [16]. It comprises three main strategies. The first strategy is Monitoring (M) of salt intake in the Malaysian population. The second strategy is to increase Awareness (A) of salt reduction practices, targeting specific groups. The third strategy is Product (P), by strengthening the mandatory salt targets for specific processed foods and voluntary reformulation. The mid-term evaluation of the policy reported that the strategies were high in trustworthiness and quality. However, the interventions have been moderately delivered and adopted by the respective organizations. It is also reported to be low in reaching the Malaysian population as the target audience [17]. One of the major barriers to implementing salt reduction was the absence of mandatory sodium content labelling on packaged foods. This hinders the monitoring, reformulation and identification of low salt food eligible for Healthier Choice Logo (HCL) recognition, as well as causes difficulties amongst consumers in choosing low salt products [18]. Nevertheless, the mandatory sodium content labelling on packaged foods was successfully gazetted in the year 2020. However, its enforcement has been postponed till January 2024, and is currently ongoing. Efforts are being conducted to monitor adherence towards enforcement.

Within the revised salt reduction policy 2021–2025, emphasis has been given to raising awareness, implementing intervention, promoting research including consumption studies and market surveys, promoting partnerships with multi-organization and food industries and monitoring the progress of salt reduction initiatives. Table 1 summarizes the comparison of activities between the two term periods of the policy.

**Table 1:** Comparison of salt reduction strategy to prevent and control NCDs for Malaysia 2015–2020 and 2021–2025

Activity	Salt reduction strategy to prevent and control NCDs for Malaysia	
	2015–2020	2021–2025
Monitoring		
Population-based survey	Among the health staff	The population-based survey to be incorporated in the National Health and Morbidity Survey-Nutrition Survey
Online Malaysian Food Composition Database (MyFCD)	Create the MyFCD database	Strengthen the MyFCD database by increasing the number of food items analysed via primary/lab analysis and items entered in the Industry Database by the food industries
		Conduct a survey to determine barriers and enablers towards salt reduction among targeted groups (e.g. out-of-home sector, B40)
Research	Research on salt intake among specific groups	Conduct a survey to determine health literacy related to salt intake among Malaysians
		Population-based survey to be incorporated in the National Health and Morbidity Survey-Nutrition Survey
Awareness		
Health promotion and education	Generally stated as health promotion and education without further clarification	Health promotion activities to increase the health literacy including reading of food labelling (to maximize benefits of mandatory salt content labelling) to specific groups
Intervention	Incorporate salt reduction interventions into *Komuniti Sihat Pembina Negara* (KOSPEN)	Use existing platforms and programs in specific target groups (i.e. preschool and school children, adolescents or young adults, adults and working population, chronic disease and elderly)
Promotion	Promotion through mass media and social media	Develop a strategic communication plan to improve health promotional activities relating to reducing salt intake
Product		
Product reformulation	Product reformulation of high-salt content processed foods	Product reformulation of pre-packaged food towards a healthier sodium level
Sodium labelling	Labelling of sodium content in processed foods	Inclusion of mandatory maximum salt/sodium level in high-risk food through/in Amendment of Food Act 1983
		Enforce the labelling of sodium content in processed food by 2022

Briefly, in the revised salt reduction policy, the monitoring of salt intake will be evaluated using urinary sodium analysis (i.e. 24-h urinary sodium or validated spot urine). In addition, the food consumption surveys on food products that have not been surveyed previously, that is, street food vendors, restaurants, school/workplace canteens, food courts, food trucks and other commercial food suppliers, should also be conducted. This monitoring will produce the outcomes of the overall impact of the strategies. The multi-stakeholder strategic communication targeting specific groups, including school children, patients with chronic disease, the low income population with household income below RM 4850 per month (approximately US$ 1026) and working adults, have also been proposed to increase awareness among the population. Furthermore, the effort to propose mandatory salt targets for specific food products that are high in salt has been highlighted.

To date, a total of 67 products have been reformulated that lowered their salt content by 2–80%. This figure achieved the target of five products per year to be reformulated with lower salt content [15]. However, this effort has merely been conducted voluntarily by the food industries. There is a need to sustain this effort by providing appropriate support. There is also a need to embark on targeted reformulation based on food categories that would give a greater impact on lowering the salt intake of the population. In addition to food reformulation, the Healthier Choice Logo (HCL) initiative that was introduced in Malaysia as a voluntary label in April 2017 has also shown some progress. Thus far, about 702 food and beverage products from more than 10 food groups contributing to salt intake of the population (e.g. pasta, biscuits, crackers, cereals, canned sardine/tuna and instant noodles) have been awarded the HCL [19]. Nonetheless, there are several other food categories with high salt content including soy sauce and other sauces yet to be awarded the HCL.

### Feedback from the salt reduction policy in Malaysia from different stakeholders

**Fig. 2:**
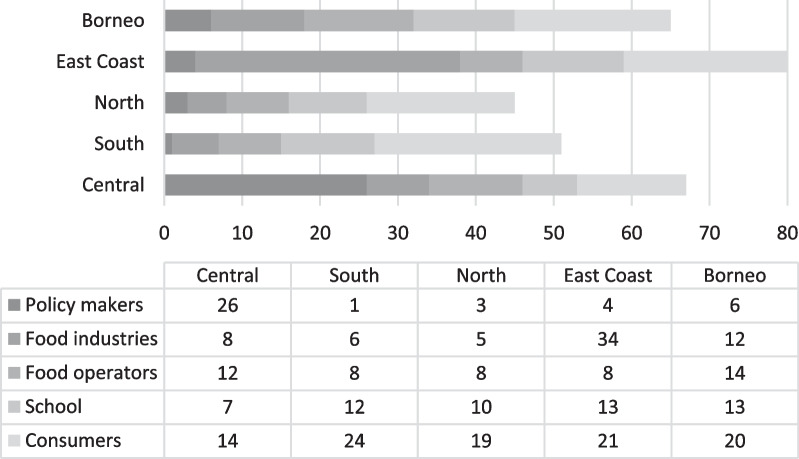
Targeted stakeholders and sample size according to region in Malaysia. Central: Kuala Lumpur, Selangor; South: Johor, Melaka, Sembilan; North: Perlis, Kedah, Penang; East Coast: Pahang, Terengganu and Kelantan; Borneo Sabah and Sarawak. [19–21]

As the salt reduction policy has already reached the mid-point of its extension term, there is a need to revisit the strategy to ensure that it is effective and sustainable. Hence, an exploratory study under the Newton Fund Impact Scheme titled ‘Developing a policy to reduce the salt content of food consumed outside the home in Malaysia’ was conducted from May 2020 to November 2022, as reported earlier [20]. This study was conducted to get feedback from different stakeholders on their perception of salt intake as well as the barriers and enablers of the ongoing salt reduction policy in Malaysia. This feedback is important to provide insights to policy-makers regarding the effectiveness and sustainability of the current strategies from different perspectives. As summarized in Fig. 2, various stakeholders (308 respondents), including policy-makers (e.g. government, non-government organizations and researchers), food industries, food operators (i.e. catering operators and street food vendors), schools (i.e. parent teacher associations and school cafeteria operators) and consumers in five regions in Malaysia participated in the study [21–23].

Findings from the exploratory study showed that most of the respondents agreed that salt intake in Malaysia is high, hence the salt reduction policy should address barriers and enablers which involve all parties in the strategies. Table 2 briefly summarizes several issues and ways to improve salt reduction in Malaysia that were suggested by the respondents.

**Table 2:** Issues and ways to improve salt reduction in Malaysia

Issue	Barriers	Enablers
Awareness	(1) Lack of awareness among food industries/operators to reformulate and produce low salt foods (2) Lack of awareness and poor behaviour among consumers towards consumption of high salt foods that influence acceptance towards lower salt foods (3) Media advertisement of unhealthy foods attract consumers, especially children, to buy unhealthy foods that are high in salt	(1) Creates awareness among food industries/operators to reduce salt in food products and prepared meals (2) Recognition given to lower salt foods/meals or premises that prepared lower salt/healthy foods to encourage reformulation and production of lower salt foods (3) Campaign that is clear, inclusive and use various communication channels to create awareness among consumers on the importance of consuming lower salt foods and restricting the advertisement of the unhealthy foods that are high in salt
Knowledge	(1) Lack of knowledge among food industries/operators on food reformulation (2) Lack of knowledge among food operators to prepare lower salt foods/meals (3) Lack of knowledge among consumers on the recommended salt intake	(1) Assist the food industries/operators in reformulation and use proper technology in food processing without compromising taste and quality (2) Provide standard guidelines and mandatory courses and trainings to food operators in food preparation (3) Educate the consumer on the recommended salt intake and emphasize the importance of reading food labelling to make informed choices. Education can also be carried out in school via health programs that will introduce school children to knowledge on healthy eating and change their attitudes and behaviours towards healthy and low salt foods
Resources	(1) Lack of funding and manpower to carry out salt reduction campaign (2) Increase of cost due to reformulation and the use of technology in production of low salt foods	(1) Provide sufficient resources (funding and manpower) to do impactful campaign (2) Financially support food industries, especially small and medium enterprises (SMEs) to carry out reformulation and use technology in food processing to reduce salt
Priority	Lack of support and prioritization for salt at the ministry and government agency level	Involvement in political drive could make government prioritize the salt reduction policy
Monitoring and enforcement	(1) Lack or absence of monitoring and enforcement will make food industries/food providers feel safe in not following the act	(1) Frequent monitoring of packed foods and foods prepared at out-of-home sector and give punishments, including withdrawing business licenses, if they do not follow the act (2) Guidelines for selling foods in school or outside the school need to be created to prevent unhealthy foods sold at these areas
Price and availability	(1) Natural flavour enhancers and salt substitutes (e.g. potassium chloride, KCl) are pricier than regular salt (2) Lower salt foods are expensive and not easily available	(1) Reduce the price of lower salt products, natural flavour enhancers and salt substitutes (2) Raise the price of regular salt and impose a salt taxation

Overall, salt reduction has received very little attention when compared with other food components such as sugar, according to some of the respondents in the study. Over consumption of sugar is the major cause of obesity and diabetes in Malaysia; thus, one of the measures taken by the government included imposing sugar tax on sweetened beverages, which came into force on 1 July 2019, and is officially known as Sweetened Beverages Excise Duty [24]. The sugar tax is expected to reduce the demand for sweetened beverages among consumers, as reported by other countries such as Thailand [25]. Sugar tax on sweetened beverages has also encouraged reformulation to reduce the sugar content in beverages to avoid being taxed, which may lead to positive health results [26].

Most researchers (food scientists) as well as non-governmental organizations (NGOs) involved in advocating about nutrition, diet and diseases related to salt interviewed in the study reported that they had lesser involvement in food reformulation and programmes related to salt reduction as compared with other food components, such as sugar. Furthermore, the food industries agreed that they could play a pivotal role in reformulating their products. Salt reduction awareness campaigns should also include reformulated products to showcase that it is feasible and possible to lower the salt content in foods. Representatives of the food industries highlighted the need for more guidance on salt targets for specific foods, and appropriate reformulation without affecting the product shelf life and customer acceptance. Food vendors and catering operators also raised concerns about consumer acceptance of lower salt meals and its effect on their business. Thus, it is essential for a salt reduction strategy for the out-of-home sectors to involve multiple stakeholders, that is, policy-makers from government agencies, researchers and non-governmental organizations, as well as engaging the food industries and other food providers (catering operators and food vendors) in increasing awareness and efforts to reduce salt content through a continuous specific strategy for the out-of-home food sector. As mentioned earlier, setting the salt target for specific food products for reformulation is urgently needed. Work has been carried out to determine the salt reduction target for soy sauce [27].

### Supplementary document for salt reduction policy of Malaysia for out-of-home sectors

The outcomes from the exploratory study have served as the basis for our team to propose a supplementary document to complement and support the current salt reduction policy. This document will enhance the current salt reduction strategies, particularly in addressing the challenges highlighted by different stakeholders, including the practicality, compliance and monitoring of the salt reduction policy, particularly for the out-of-home sector. A further purpose of this document is to strengthen the Monitoring, Awareness and Products strategies of the Salt Reduction Strategy 2021–2025, particularly targeting reduction of salt in processed foods and foods that are prepared in the out-of-home sectors. This will ensure that the goal of achieving a 30% reduction of global target by the year 2025 with the aim of reducing the burden of NCDs in Malaysia and emphasizing the out-of-home sector (MySaltOH) can be achieved. This proposed supplementary document will promote, educate and collaborate with all relevant stakeholders to reduce salt intake among the Malaysian population. Several objectives were highlighted in this supplementary document, including:

1. To strengthen national policies and plans for the prevention and control of NCDs through salt reduction initiatives in the out-of-home sector via a consortium involving multiple stakeholders.
2. To raise the level of awareness of the issue of salt consumption in the out-of-home sector in the prevention of NCDs through specific and targeted monitoring and intervention involving multiple agencies.
3. To engage and empower food industries to reduce salt in processed food products through voluntary food reformulation in collaboration with policy-makers and researchers.
4. To engage and empower food vendors in providing healthier food with a lower salt content for consumers in the out-of-home sector in collaboration with policy-makers and other agencies.
5. To recognize and acknowledge efforts towards salt reduction in the out-of-home sector through a sustainable and cost-effective approach.

### Action plan for salt reduction policy of Malaysia for out-of-home sectors

The action plan of the salt reduction policy of Malaysia for out-of-home sectors adopted the M-A-P Strategies as outlined by the Ministry of Health Malaysia.

#### Monitoring strategy (M)

In this strategy, it is suggested that future nationwide studies [i.e. the National Heath Morbidity Survey (NHMS) or the MyCoSS study] include monitoring of salt intake in other population groups, including adolescents and children, either using 24-h urinary sodium or spot urine measurement. The current salt reduction policy does not include adolescents and children, thus it is also beneficial to determine the food habits of these age groups. In addition to Knowledge, Attitude and Practice (KAP) assessment of the population, a specific KAP evaluation on salt intake among a sub-sample of food industries and food providers such as catering operators and food vendors should be conducted. This will ensure that the monitoring strategy will reach more food providers. Besides that, a continuous regular impact assessment on the success of the salt reduction promotion strategies, market survey and surveillance of food products’ compliance towards salt labelling/mandatory salt targets is also proposed. This will provide a more comprehensive database for consumers to make informed choices as to foods that have reduced salt content. Another recommended strategy includes the guidelines to reduce salt content in fast food restaurants. It is suggested that digital technology be adopted to assist government agencies in monitoring adherence towards some of these strategies. For example, a digital platform could be developed to allow consumers to give direct feedback of non-compliance of food industry towards labelling guideline.

#### Awareness strategy (A)

As shown in Table 2, there is a knowledge gap among food industries and food providers regarding salt and ways to reduce salt in foods. It is recommended to increase awareness, particularly targeting the food industries and food providers and the consumers, via Training of the Trainer (ToT) on salt reduction with involvement of agencies other than MoH. Additionally, it is recommended to integrate and emphasize healthy eating concepts into food handling courses. The current initiatives by the government to encourage provision of healthy food should be promoted widely. For example, at the moment there are 109 cafeterias being certified as healthy [28], particularly at hospitals and health clinics, and 2997 food premises being awarded with BeSS. BeSS recognition is the "Clean, Safe and Healthy" recognition, given to food premises operators to encourage food premises operators to provide safe and healthy food to customers  [29]. In addition, salt reduction intervention at targeted settings such as schools, universities and workplaces are recommended in collaboration with multiple stakeholders. Provision of recognition and incentives to food industries/food providers and others that have successfully implemented salt reduction strategies is proposed to accelerate the rate of success.

#### Product strategy

To reduce salt in products, several strategies have been highlighted. These include food reformulation, mandatory legislation of salt labelling, enforcement of legislation for maximum salt allowed for 14 food categories (Table 3), improvement and standardization of the salt term used in nutritional labelling (as there was confusion regarding the terminology among many respondents in our study), encouragement of the provision of healthy food options and recommendation of specific guidelines for reducing salt in the out-of-home food sectors.

**Table 3:** Proposed maximum sodium level of 14 food categories

No	Foodstuff category
1	Soya sauce or soya bean sauce or *kicap* (e.g. light/salty/sweet)
2	Tomato ketchup or tomato sauce or tomato catsup
3	Chili sauce
4	Canned fish
5	Canned meat (includes products that fall under Food Regulation Standard 149 and Standard 151)
6	Manufactured meat (e.g. sausage, meat burger, corned, cured, pickled salted meat)
7	Instant noodles
8	Fish snacks (e.g. *Keropok lekor*)
9	Soup
10	Soup stock
11	Processed cheese
12	Butter
13	Margarine
14	Prepares cereal food (breakfast cereal)

Source: Ministry of Health [30]

##### Operationalization of the strategy

The plan of action for salt reduction policy of Malaysia for out-of-home sectors and roles of different ministries or government agencies is presented in Table 4.

**Table 4:** Plan of action for salt reduction policy of Malaysia for out-of-home sectors

Strategy	Activity	Indicators	Targets	Lead agency
Monitoring	Surveillance of Knowledge, Attitude and Practice (KAP) related to salt intake of the population (consumers and parent teacher associations)	A survey conducted every 5 years	Baseline study in 2024	Institute for Health Behavioural Research (IHBR)
	Surveillance of salt consumption pattern of the population via urine sodium level (24-h urine) and dietary intake	A survey conducted every 5 years	To be incorporated in NHMS 2024 & 2029	Institute for Public Health (IPH)
	A study of salt consumption pattern of children and teenagers in school or clinic setting	A survey conducted every 5 years	Pilot study to be conducted by 2027 under 13th Malaysia Plan	Institute for Public Health (IPH)
	A KAP study of salt intake in food vendors, catering operators and food industries	A survey conducted every 5 years	The first survey to be conducted by 2026 under 13th Malaysia Plan	Institute for Health Behavioural Research (IHBR)
	A market survey on products (e.g. processed foods, fast foods) to determine the compliance towards achievement of salt reduction strategies: • The salt (sodium) content in processed foods	Number and percentage of products with: • Salt (sodium) content in processed foods	To be conducted by 2027	Disease Control Division
	To monitor complaints regarding the non-compliance of sodium labelling via *Sistem Pengurusan Aduan Awam* (SISPAA), MOH (towards empowering the utilization of digital platform in monitoring salt reduction strategies)	Number of actions taken on the report of sodium labelling non-compliance	All reports on non-compliance of sodium labelling should be acted upon accordingly	Food Safety and Quality Division
Awareness	To conduct workshop or training of the trainers’ programme about salt reduction among officers from ministries other than MOH	Number of ToT/workshops conducted	At least once per year	Disease Control Division
	To conduct targeted interventions at specific settings such as schools, universities, workplaces and B40’s community (e.g. collaborate with KOSPEN, KOSPEN WOW)	Number of interventions per year	Minimum one intervention per setting per year	Disease Control Division
Product	Product Reformulation & Guidelines			
	To conduct food reformulation on 14 categories of foods with maximum salt targets	The number of product reformulated	All 14 categories of food have been reformulated by 2025	Disease Control Division
	To recommend specific guidelines to reduce salt content in fast food restaurants by incorporating the Best Practices for the Fast-Food Industries. (e.g. salt served separately from French fries)	Preparation of guidelines	To be published by 2025	Nutrition Division
	To propose maximum sodium content of 2000 mg/day in daily meals in hospital (in patient)	Number of hospitals	To increase at least 5% per year	Medical Development Division
	Strengthen and enforce the law			
	To propose to include salt content (‘salt’ term to be used) on the labelling in addition to sodium	Compulsory labelling	2030	Disease Control Division
	To enforce compliance towards mandatory salt (sodium) labelling in foods	Number of products labelled for salt/sodium content	100% of products examined have a sodium label by 2024	Food Safety and Quality Division
	To support the gazettement of legislation for maximum salt target for 14 food categories	Number of gazette products	20% of the 14 food categories are gazetted by 2027	Food Safety and Quality Division
	To propose HCL salt target for soy sauce products according to type: (a) ≤ 9% of salt (≤ 4 g of sodium) and ≤ 15 g of sugar for salty soy sauce and (b) ≤ 7% of salt (≤ 3 g of sodium) and ≤ 30 g sugar for sweet soy sauce	Proposal of HCL salt target for salty and sweet soy sauces	HCL salt target will be implemented by 2024	Disease Control Division
	To process the Amendment of Malaysian Food Act 1983 (Food Regulation 1985) (1) Soy sauce shall contain not less than 0.4% total nitrogen and 7% salt (2) Maximum salt content: 11% (4.4 g sodium)	Gazettement of the Amendment	Amendment is gazetted by 2027	Food Safety and Quality Division
	Recognition			
	To propose HCL as one of the criteria for procurement specification of hospital food items	Number of items	To increase at least 5% per year	Procurement & Privatization Division
	To give recognition and incentives to out-of-home sectors’ food providers such as food industries, food vendors, caterers and other agencies that successfully implement salt reduction strategies e.g. Food industries – premium products; Food vendors – *Kantin Contoh*, *Sekolah Sihat*, subsidies to canteen, contract renewal Media – healthy food hunting show (*Jalan-Jalan Cari Makan Sihat*)	Number of recognitions according to out-of-home food sector category	One for each category per year	Disease Control Division

Possible roles of other governments and non-governmental agencies in salt reduction strategy for out of sector were also discussed and are presented in Tables 5 and 6, respectively. The listed roles could be referred by these agencies to take an active part in the salt reduction strategy to ensure its sustainability.

**Table 5:** Government agencies that have major possible roles in operationalizing the salt reduction strategies

Ministry	Possible roles in the salt reduction strategy
Ministry of Education/higher education	• Incorporating salt education components in schools’ healthy eating curriculum • Facilitating the implementation of salt reduction initiatives/programme in schools or higher learning institutions • Creating a health-promoting environment (e.g. healthy cafeteria or canteen, school meal programme)
Ministry of Communication and Multimedia	• Facilitating promotion of salt reduction programme and low salt foods via electronic media, that is, television, radio and social media, and printed media, that is, newspapers, for wider coverage
Ministry of International Trade and Industry	• Facilitating the development of product reformulation among the small and medium food industries • Assisting and promoting the exportation of local products with low or reduced sodium content • Accelerate the use of digital technology and salt-related innovation, such as reformulation • Financial support for marketing of low salt food products globally
Ministry of Domestic Trade, Cooperatives & Consumerism	• Promotion of healthier or low/reduced salt foods/beverages as an affordable alternative for Malaysians • Facilitate the implementation of fiscal measures (i.e. subsidy plan) and regulations related to unhealthy foods/beverages • Financial support in subsidizing healthier food products
Ministry of Agriculture and agro-based industry	• Promote the eating of fruits and vegetables to increase potassium intake and reduce salt intake • Increase the availability of fresh vegetables and fruits to Malaysians at affordable prices to increase consumption • Accelerate the use of digital technology and salt-related innovation, such as reformulation • Financial support for reformulation and marketing of low salt food products
Attorney General’s Office	• Approval of proposed legislation or amendment on Food Act

**Table 6:** Roles of other agencies outside of health

Stakeholder	Possible roles in the Salt Reduction Strategy
Food industries, catering operators, food vendors	• Reformulation of food products with reduced and low sodium content in line with the Healthier Choice Logo requirements • Reformulation of food products by gradually reducing the amount of salt added, particularly to meet the salt targets • Contributing to the MyFCD database of reformulated food with reduced and low sodium content • Labelling of food with sodium level following the mandatory sodium labelling by the Food Act
Researcher/academia	• Conduct translational research to reduce salt in foods, that is, survey on salt content in processed or cooked foods, salt intake survey in targeted group, intervention study to reduce salt in targeted group, knowledge, attitude and practice (KAP) on salt reduction and its relationship to hypertension and cardiovascular diseases • Collaboration with food industries to reformulate foods that are high in salt
NGOs, for example, MyWASSH Malaysian Society for World Action on Salt, Sugar and Health	• Awareness and education to general population • Advocate target groups on salt reduction programs and initiatives
Media (i.e. famous/celebrity chefs or influencers)	• Consumer awareness about reduced and lower salt foods • Promotion of lower salt foods • Promotion of safe salt alternative and substitutes • Educate the public to reduce salt in their food preparation
Schools, parent teacher associations and consumers	• Both parents and teachers are actively sought to take part in the salt reduction campaign • Gradually reduce salt intake by reduction of consumption of high salt foods and reduce the use of salt and sauces during cooking whether at home (by parents) or at school (by food providers) • Read the food labelling and chose foods with lower salt content • Request less salt when eating outside
	• Promote the consumption of low sodium foods

##### Monitoring and evaluation

The indicators and targets that have been presented in Table 3 should be monitored by the lead institution, that is, the MoH, which monitors three scopes, including policy, advocacy and research. For the long term, the current indicators and targets outlined by the MoH, which is in line with the global target of the WHO [8], should be followed (Table 7).

**Table 7:** Long-term indicators and targets

Long-term outcome indicators	Target (2025)
Average salt intake of the adult population	Less than 6.0 g of salt per day
Risk of premature mortality due to hypertension, cardiovascular diseases and stroke	25% relative reduction of the prevalence from 2010 (baseline)

Ministry of Health [18]

## Conclusions

This proposed supplementary document for Salt Reduction Policy of Malaysia at out of home sectors with the M-A-P strategies would strengthen the current Salt Reduction Policy 2021–2025. In collaboration with multiple stakeholders, it could also reach a wider target population, thus, several steps are proposed to strengthen the salt reduction interventions:

1. Emphasized integration of different elements for salt reduction that target all ages of the general population is needed and could be facilitated by stakeholders to create impactful and sustainable strategies.
2. A comprehensive monitoring system with random inspection of ready-to-eat processed food and out-of-home dining food needs to be established to evaluate the progress of the salt reduction initiatives taken up by the food industries and food providers.
3. Effective communication channels should be adopted to convey and reinforce salt reduction messages to the public as part of the continuous public education and awareness on the salt reduction strategies.
4. Interventions on salt reduction should be conducted with ongoing evaluation to be able to adapt throughout the process.

All of these steps will effectively address the gaps identified in the 2015–2020 policy. It is hoped that this proposed supplementary document could be an aid for the Ministry of Health Malaysia and other relevant ministries as well as related agencies in the salt reduction strategy. It also will provide guidance to the food industries to execute the salt reduction strategy for the out-of-home sector. Despite the long-term target of 5.5 g salt intake (30% reduction) to be achieved by 2030, it is hoped that the initial achievement of 6 g of salt intake (25% reduction) by 2025 wil be accomplished, as outlined in this supplementary document.

## Data Availability

All data were treated as confidential and not publicly available but could be disclosed through the corresponding author on a reasonable request.
